# Characterization and Transcriptome Studies of Autoinducer Synthase Gene from Multidrug Resistant *Acinetobacter baumannii* Strain 863

**DOI:** 10.3390/genes10040282

**Published:** 2019-04-08

**Authors:** Chung-Kiat Ng, Kah-Yan How, Kok-Keng Tee, Kok-Gan Chan

**Affiliations:** 1 Department of Medical Microbiology, Faculty of Medicine, University of Malaya, Kuala Lumpur 50603, Malaysia; ngchungkiat@siswa.um.edu.my (C.-K.N.); k2tee@um.edu.my (K.-K.T.); 2 Division of Genetics and Molecular Biology, Faculty of Science, Institute of Biological Sciences, University of Malaya, Kuala Lumpur 50603, Malaysia; kyhow2@um.edu.my; 3 International Genome Centre, Jiangsu University, Zhenjiang, China

**Keywords:** *Acinetobacter baumannii*, quorum sensing, transcriptomic, RNA-Seq, recombineering, mutagenesis

## Abstract

Quorum sensing (QS) is a cell-to-cell communication system that uses autoinducers as signaling molecules to enable inter-species and intra-species interactions in response to external stimuli according to the population density. QS allows bacteria such as *Acinetobacter baumannii* to react rapidly in response to environmental changes and hence, increase the chances of survival. *A. baumannii* is one of the causative agents in hospital-acquired infections and the number of cases has increased remarkably in the past decade. In this study, *A. baumannii* strain 863, a multidrug-resistant pathogen, was found to exhibit QS activity by producing *N*-acyl homoserine lactone. We identified the autoinducer synthase gene, which we named *abaI,* by performing whole genome sequencing analysis of *A. baumannii* strain 863. Using high resolution tandem triple quadrupole mass spectrometry, we reported that *abaI* of *A. baumannii* strain 863 produced 3-hydroxy-dodecanoyl-homoserine lactone. A gene deletion mutant was constructed, which confirmed the functionality of *abaI*. A growth defect was observed in the QS-deficient mutant strain. Transcriptome profiling was performed to determine the possible genes regulated by QS. Four groups of genes that showed differential expression were discovered, namely those involved in carbon source metabolism, energy production, stress response and the translation process.

## 1. Introduction

Quorum sensing (QS) is a sophisticated cell-to-cell communication system that involves the production, detection and response to signaling molecules known as autoinducers. QS system allows bacteria to detect its populations density in response to the concentration of autoinducers [1]. There are three steps involved in the QS system [1]. First, the autoinducers are produced in the cells. Second, these autoinducers either diffuse or are actively transported to the extracellular environment and their concentration increases as the population density increases. Third, when the concentration of the autoinducers goes beyond the minimal threshold for detection, the autoinducers bind to the intracellular cognate receptors in the cells. Autoinducer-bound receptors act as transcriptional factors and induce cell signaling cascades resulting in a change in gene expression on a population basis [2]. Alterations in gene expression can trigger phenotypic changes as a response to the environmental changes. These phenotypic changes include biofilm formation [3], antibiotic resistance [4], toxin secretion [5] and conjugation [6,7]. There are various autoinducers and their respective receptors are used by different bacterial species. For instance, the QS system in *Vibrio fischeri* involves two genes, autoinducer synthase, *luxI* and autoinducer receptor, *luxR* and autoinducer, and *N*-acyl homoserine lactones (AHLs) [8]. Many Gram-negative bacteria including *Acinetobacter baumannii* possess *luxIR* homologues and utilize AHLs as autoinducers [9].

*A. baumannii* is a Gram-negative, catalase producing, non-fermenting and strictly aerobic coccobacilli [10]. This bacterium has become increasingly important because of its frequent association with nosocomial infections, especially among immunocompromised patients [11]. It was once coined the nickname “Iraqibacter” due to many infections found in wounded soldiers that served in Iraq [10]. There was a misconception that *A. baumannii* can be isolated from various environments [12]. While this is true for the genus which contributed to the misconception, the natural reservoir of *A. baumannii* is still remained to be identified [13,14,15]. However, the hospital environment has become an important reservoir [13,14,16]. In addition, *A. baumannii* has acquired resistance towards a wide range of antibiotics and recent studies suggested that the prevalence of multidrug-resistant *A. baumannii* is still on the rise in many countries [17]. In 2017, the World Health Organization (WHO) has included *A. baumannii* as one of the most dangerous pathogens hit-list and hence, the highest priority in new antibiotic development [18]. Being able to persist in hospital environment and to defend themselves against multiple antibiotics show that *A. baumannii* has an amazing capability to survive and adapt in a harsh environment.

Since the QS system is used by various bacterial species to react against changes in the environment, it is of interest to elucidate the genes regulated by this cell-to-cell communication system. Transcriptomic profiling fits well in this study as QS controls a wide range of genes. Transcriptomic profiling previously employs hybridization-based microarray. However, as next-generation sequencing (NGS) technology has improved, RNA-sequencing (RNA-Seq) has become more common in transcriptomic studies. This is because RNA-Seq provides three advantages over hybridization-based microarray platforms: (i) detection of novel transcripts, (ii) higher resolution data, and (iii) a higher dynamic range [19,20]. Hence, whole genome sequencing and RNA-Seq could work hand in hand to provide a more comprehensive way to elucidate the expression network revolved around QS in *A. baumannii.*

In this study, we characterized the autoinducer synthase gene, *abaI*, in a multidrug-resistant *A. baumannii* strain 863 and the genes regulated by QS using mutagenesis and transcriptome analyses. With verification of the synthase activity, it provides a platform to study the regulatory role of the AHLs on the virulence and unknown genetic traits of this pathogenic isolate. With further insights of the role of the secreted AHLs, it facilitates the antimicrobial strategies to attenuate bacterial virulence among *Acinetobacter* sp.

## 2. Materials and Methods

### 2.1. Bacterial Strain and Growth Conditions

*A. baumannii* strain 863 was obtained from the culture collection of a local diagnostic lab. The strain was cultured in Luria-Bertani (LB) medium. Table 1 shows the bacteria strains and plasmids used in this study.

### 2.2. Whole Genome Sequencing (WGS), Assembly and Annotation

The genomic DNA from strain 863 was extracted using the Masterpure DNA Purification Kit (Epicentre, Madison, WI, USA) according to the manufacturer’s protocol. The quality of the genomic DNA was assessed using a Nanodrop Spectrophotometer (Thermo Fisher Scientific, Waltham, MA, USA). The extracted DNA was used in Nextera Library Preparation Kit (Illumina San Diego, CA, USA). The prepared library was sequenced using 100 bp × 2 cartridge in HiSeq 2500 High Throughput Sequencer (Illumina) on rapid run mode. The quality of the sequenced data was assessed using FastQC software [22]. The sequenced data were then trimmed and assembled using CLC Genomic Workbench (V7.5; Qiagen, Hilden, Germany). Following this, the assembled sequence was annotated by the National Center for Biotechnology Information (NCBI) Prokaryotic Genome Automatic Annotation Pipeline (PGAAP) [23] and RAST [24].

### 2.3. Autoinducer Synthase Identification and Bioinformatics Analysis

The nucleotide sequence generated from the WGS which was annotated as autoinducer synthase (*abaI*) was used to compare with GenBank databases via BLASTX. Eleven protein sequences of autoinducer synthases from *Acinetobacter* species were selected. These sequences were aligned using ClustalW and a phylogenetic tree was generated via Maximum Likelihood method using MEGA 7 [25]. The algorithm applied the Jones-Taylor-Thornton (JTT) matrix-based model [26] and to provide confidence estimation, 1000 bootstrap replications were used to construct the phylogenetic tree. On the other hand, the putative promoter sequences were identified using BPROM webtool [27]. All parameters were set to default settings, unless specified.

### 2.4. AHL Extraction and Its Identification Using Mass Spectrometry (MS)

One millimeter of an overnight culture of strain 863 was subcultured into 100 mL LB broth buffered to pH 6.5 with 50 mM of 3-[*N*-morpholino] propanesulfonic acid (MOPS) to prevent the AHLs from being degraded in a basic medium [28]. The culture was then incubated overnight at 37 °C with agitation at 230 rpm. The culture supernatant was obtained by centrifugation and subsequently extracted three times with equal volume of acidified ethyl acetate (0.1% v/v glacial acetic acid in ethyl acetate). The extracts were dried in a fume hood before resuspended with 1 mL of acidified ethyl acetate and dried again inside fume hood. The final dried extracts were reconstituted in 1 mL of acetonitrile. The insoluble solids in the extracts were removed using a syringe filter. AHL identification was done using high-resolution tandem triple quadrupole mass spectrometry (LCMS/MS; Agilent 1290 Infinity LC coupled with Agilent 6490 Triple Quadrupole LC/MS system, Agilent Technologies Inc., Santa Clara, CA, USA).

### 2.5. Construction of Abai Deletion (ΔabaI:Km) Strain

**Figure 1:**
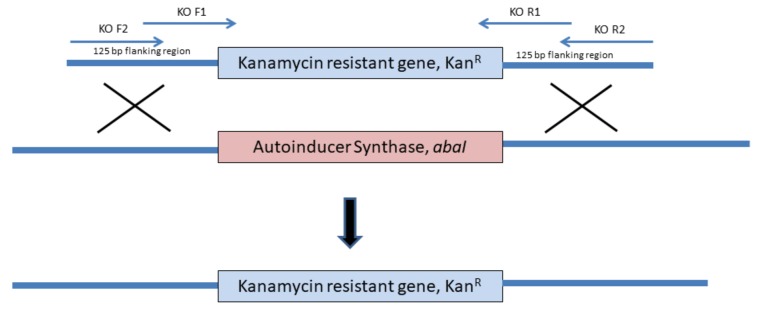
Schematic diagram of the KO cassette construction and *abaI* deletion process in the cell. Two pair of primers were used to create a selectable marker with 125 bp flanking regions from upstream and downstream of *abaI*. The gene *abaI* was replaced with kanamycin resistant gene as a selectable marker with Re_AB_ system from pAT04 transformed into *A. baumannii* 863 earlier.

An autoinducer synthase (*abaI*) deletion mutant was constructed as described by Tucker et al. [21]. In brief, plasmid pKD4 (Table 1) was used as a template to amplify the kanamycin resistance gene (Km) using polymerase chain reaction (PCR). Two pairs of primers were used to construct the knockout (KO) cassette Table 2. KO F1 and KO R1 primers were used to amplify the intermediate KO cassette. The amplicon was gel-purified and used as a template for a second PCR. In the second PCR, KO F2 and KO R2 primers were used to construct the final KO cassette with Km and the resultant amplicon was flanked by 125 bp upstream and downstream of *abaI* Figure 1) This PCR product (KO cassette) was gel purified and used in transformation. The plasmid pAT04 Table 1 was transformed into strain 863 through electroporation and selected via LB agar supplemented with tetracycline (10 µg/mL, final concentration). Strain 863 harboring pAT04 was confirmed with PCR using primer Screen pAT04 F and Screen pAT04 R. *A. baumannii* strain 863 harboring pAT04 was cultured in 50 mL LB broth containing 10 µg/mL of tetracycline to maintain the plasmid for 45 min before induction with isopropyl β-D-thiogalactopyranoside (IPTG (2 mM, final concentration). The induced culture was grown until the OD_600_ reached 0.4. Next, the culture was incubated on ice for 30 min before washing three times with ice-cold 10% (v/v) glycerol and concentrated 250-fold. Twenty microliters of these washed cells were transformed with 5 µg of KO cassette via electroporation. The transformants were then incubated for 4 h at 37 °C and plated on LB agar supplemented with 30 µg/mL kanamycin. The transformant colonies (mutant strain, Δ*abaI*:Km) were verified using PCR (Screen KO F and Screen KO R as primers) and the resulting mutant loss of its AHL production was confirmed with LCMS/MS. Finally, the desired mutant was sub-cultured sequentially to enable the loss of pAT04. A PCR was then performed to confirm the loss of pAT04.

### 2.6. Growth Curve Analysis

Both overnight cultures of the wild type and the mutant strains were inoculated in a 100 mL fresh LB broth, and the cell density was adjusted to OD_600_ = 0.001. The cultures were grown in a shaking incubator at 37 °C with shaking at 230 rpm. The cell density (OD_600_) was measured every 30 min until at least three readings indicated stationary phase. The experiment was performed with three biological replicates.

### 2.7. RNA-Seq Library Preparation

Both overnight bacterial cultures of the wild type and the mutant strains were sub-cultured into 100 mL LB broth at OD_600_ = 0.001 and grown at 37 °C until late log phase (OD_600_ reached 2.75 for wild type, and 2.25 for Δ*abaI*:Km) was achieved. The harvested cells were stored in RNAprotect Bacteria Reagent (Qiagen) according to the manufacturer’s instructions until RNA extraction was performed. The total RNA extraction was performed using Nucleospin RNA kit (Macherey-Nagel, Düren, Germany). The ribosomal RNA (rRNA) was removed from total RNA using Ribo-Zero rRNA Removal Kit (Bacteria) (Illumina). Finally, the rRNA depleted RNA was used as the input for ScriptSeq v2 RNA-Seq Library Preparation Kit (Illumina). The libraries obtained were sequenced using 75 × 2 cartridge on MiSeq Sequencer (Illumina). This analysis was performed with three biological replicates.

### 2.8. RNA-Seq Bioinformatics Analysis

The quality of the sequencing data was assessed with FastQC [22]. The assessed RNA-Seq sequencing data were trimmed, assembled and mapped using CLC Genomics Workbench (V 7.5; Qiagen). Then, the assembled reads were converted into BAM format and counted using HTSeq-count script from HTSeq [29]. Following this, a differential expression (DE) analysis was performed using DESeq2 [30] and a heatmap was generated. Genes with Log_2_ fold change (Log_2_FC) ≥ $\left|2\right|$ and the adjusted *p*-value of < 0.05 were considered as genes with DE.

### 2.9. Accession Numbers

The whole genome sequence of *A. baumannii* strain 863 was submitted to GenBank under accession number LZTF00000000. The RNA-Seq dataset has been deposited to Gene Expression Omnibus (GEO) under accession number GSE120346.

## 3. Results

### 3.1. Identification and Phylogenetic Analysis of Autoinducer Synthase Gene

The whole-genome sequencing of strain 863 was performed using a HiSeq 2500 High Throughput Sequencer. The genome sequence was assembled into 65 contigs consisting of 3.812 Mb. A total of 3495 coding DNA sequences (CDS) was predicted by PGAAP. The sequence has been deposited at GenBank under accession no. LZTF00000000.

**Figure 2:**
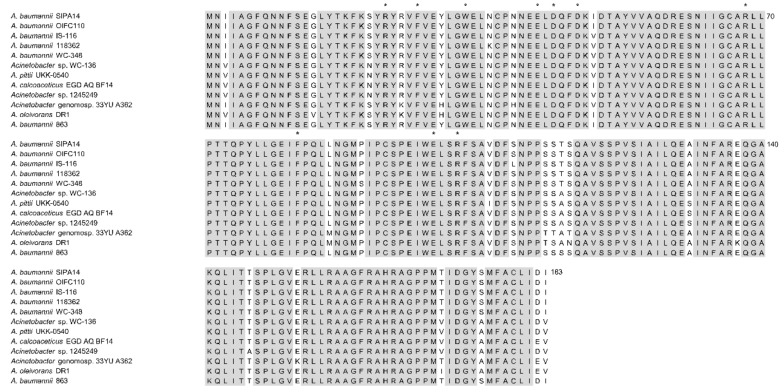
Multiple sequence alignment of autoinducer synthase protein sequence of strain 863 and other *Acinetobacter* species. Identical residues are highlighted in grey. All 10 invariant residues in autoinducer synthase homologues are labelled with asterisks. National Center for Biotechnology Information (NCBI) accession numbers: *A. baumannii* SIPA14 (OXU71347), *A. baumannii* OIFC110 (EKL58643), *A. baumannii* IS-116 (EKA70172), *A. baumannii* 118362 (EXA85498), *A. baumannii* WC-348 (EKU52127), *Acinetobacter* sp. WC-136 (EKU68177), *A. pittii* UKK-0540 (ODI99480), *A. calcoaceticus* EGD_AQ_BF14 (OBA11519), *Acinetobacter* sp. 1245249 (EXH33584), *Acinetobacter* genomosp. 33YU A362 (ONN53459), *A. oleivorans* DR1 (ADB80056), *A. baumannii* 863 (WP_029424600).

**Figure 3:**
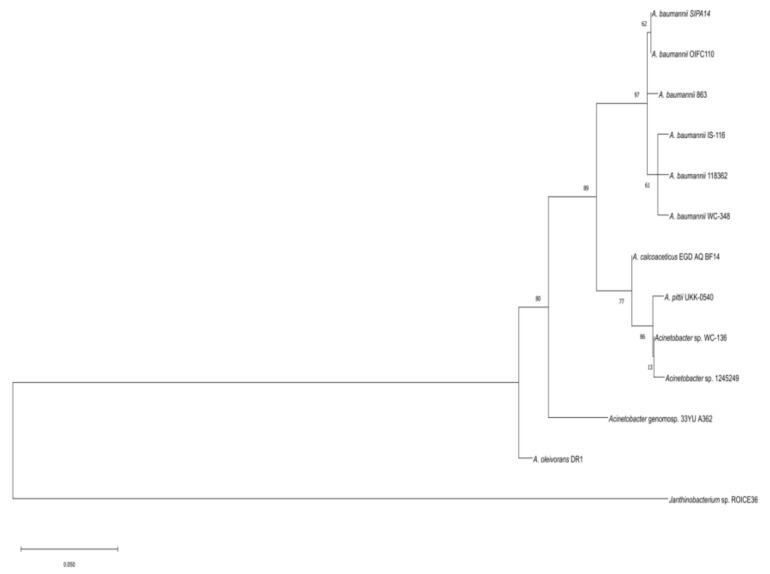
Phylogenetic tree of autoinducer synthase from strain 863 and other *Acinetobacter* species. The tree was generated using maximum likelihood method based on the Jones-Taylor-Thornton (JTT) matrix-based model. The numbers at the node show the bootstrap values as a percentage of 1000 bootstrap replications. Autoinducer synthase from *Janthinobacterium* sp. ROICE36 (WP_102124182) was used as the outgroup in this tree. NCBI accession numbers: *A. baumannii* SIPA14 (OXU71347), *A. baumannii* OIFC110 (EKL58643), *A. baumannii* IS-116 (EKA70172), *A. baumannii* 118362 (EXA85498), *A. baumannii* WC-348 (EKU52127), *Acinetobacter* sp. WC-136 (EKU68177), *A. pittii* UKK-0540 (ODI99480), *A. calcoaceticus* EGD_AQ_BF14 (OBA11519), *Acinetobacter* sp. 1245249 (EXH33584), *Acinetobacter* genomosp. 33YU A362 (ONN53459), *A. oleivorans* DR1 (ADB80056), *A. baumannii* 863 (WP_029424600).

From the annotations by PGAAP, an open reading frame of 552 bp (A9801_RS12630) was predicted to encode the AHL synthase protein (WP_029424600), which is 183 amino acids in length. To further verify the identity of this gene, its nucleotide sequence was searched against NCBI database using BLASTX program. The result revealed that the sequence is a homologue to AHL synthase and autoinducer synthase from other *A. baumannii* strains with similarity exceeded 95% identical residues. Multiple sequence alignment analysis further showed that these sequences exhibit a high degree of similarity between various *Acinetobacter* species, particularly the 10 conserved amino acids signature to autoinducer synthase homologue protein (Figure 2). A phylogenetic tree was constructed based on the protein sequences of autoinducer synthase from 11 *Acinetobacter* strains (Figure 3). The putative *abaI* gene from strain 863 was grouped with other *A. baumannii* strains with a bootstrap value of 97%. The protein sequence from the putative *abaI* was analyzed using InterProScan [31]. Results showed that the amino acid sequence matches with protein family from autoinducer synthase and possessed acyl-CoA-*N*-acyltransferase domain, a signature structural domain for autoinducer synthase.

**Figure 4:**
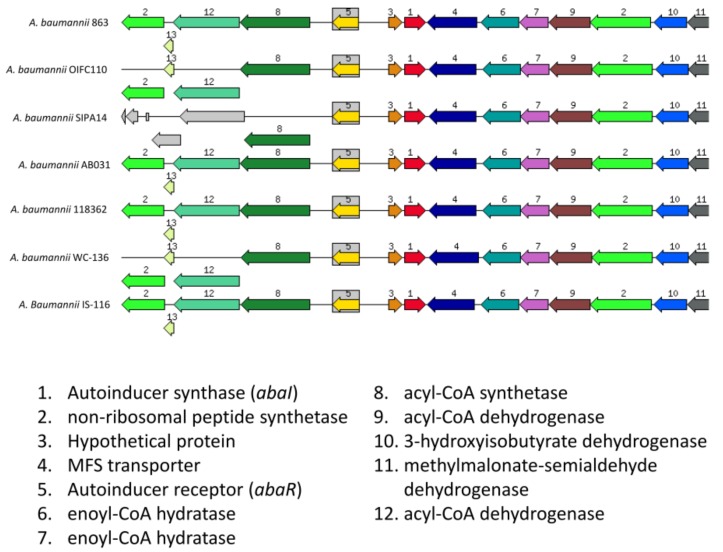
The organization of gene cluster of *abaI*. The organization of *abaI* in strain 863 and other *A. baumannii* strains.

**Figure 5:**
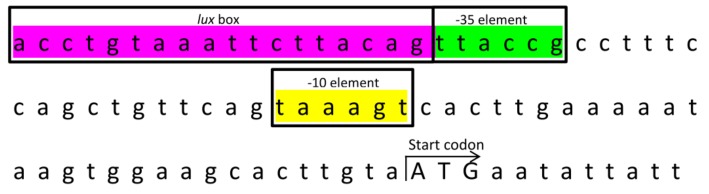
Schematic diagram of the upstream region of *abaI*. The start codon of the gene is labelled in uppercase. Promoter elements were highlighted in colors. Yellow, −10 (Pribnow box); Green, −35; Magenta, *lux* box.

When we analyzed the organization of gene cluster of abaI, we found that the gene clusters demonstrated conserved variation when compared to other *A. baumannii* strains except *A. baumannii* SIPA14 (Figure 4). To further analyze the flanking regions of this *abaI* gene, we first searched 200 bp upstream of *abaI* using BPROM to determine the presence of a promoter in this region. BPROM analysis predicted a putative −10 element (Pribnow box) sequence, TAAAGT and a −35 element sequence, TTACCG. These sequences are located at 30 and 53 nucleotides upstream of *abaI,* respectively (Figure 5). Besides, both element sequences are 17 nucleotides apart which are found to be an optimum spacing proposed by Hawley and McClure [32]. Subsequently, we aimed to identify the “lux box” which is palindromic in nature and found in other AHL producing bacteria [33,34,35,36]. We found a palindromic sequence (CTGTAAATTCTTACAG) at 59 nucleotides upstream of start codon site could be the putative lux box. This sequence is the binding site for LuxR homologue protein, designated AbaR.

### 3.2. Verification of Autoinducer Synthase Gene through Mutagenesis

**Figure 6:**
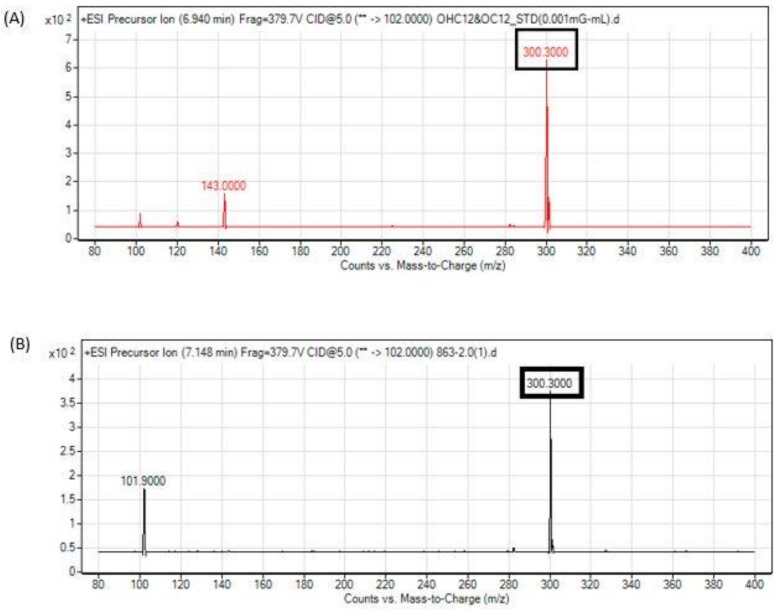
Mass spectrometry analysis shows the presence of a long chain AHL, hydroxy-C12 in the spent culture supernatant of strain 863. (**A**) Mass spectra of synthetic hydroxyl-C12 and (**B**) hydroxyl-C12 from strain 863. A peak at *m*/*z* 300.3 was detected which indicated the presence of hydroxyl-C12.

Next, we determined the authenticity of the gene with a two-part functional study experiment. First, we determined the production of AHL in strain 863. This involved AHL extraction from an overnight culture of strain 863 and these extracts were subjected to high-resolution mass spectrometry analysis to detect the presence of AHL. The LCMS/MS mass spectra showed the presence of a long chain AHL (3-hydroxy-dodecanoyl-homoserine lactone, hydroxyl C12 in short) in the spent culture supernatant of strain 863 (Figure 6). We then proceeded to confirm the putative AHL synthase gene is indeed the gene responsible for the AHL production in strain 863. This was done by replacing the putative *abaI* gene with a kanamycin resistant cassette via a recombineering method. Cell-free extracts from overnight cultures of the mutant strains were then again subjected to LCMS/MS analysis. Mass spectrometry analysis confirmed the absence of AHL in the mutant strain, therefore verified that the putative *abaI* gene was responsible for the production of AHL in strain 863.

### 3.3. Growth Curve Analysis

**Figure 7:**
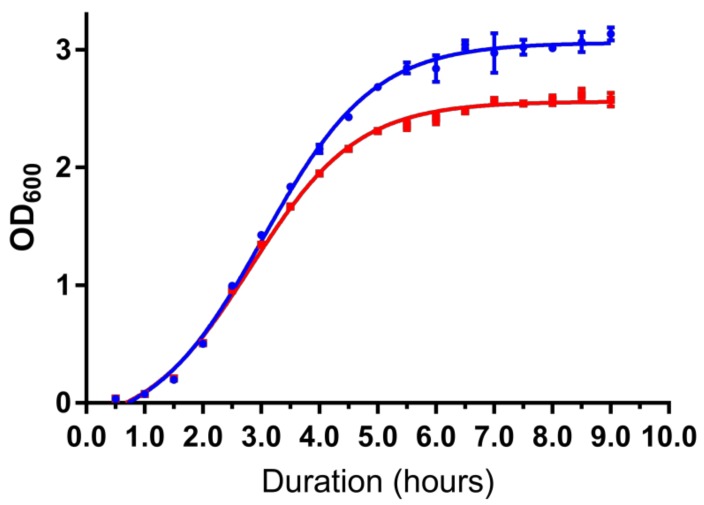
Growth curve analysis shows QS-deficient mutant suffered growth defect. Mutant strain (red line) has significantly lower cell density compared to the wild type (blue line). Data are presented as the mean (± S.E.) of three independent biological replicates.

A growth curve analysis was performed to determine the difference in growth rate between the wild type and the mutant strains (*A. baumannii* strain 863 Δ*abaI*:Km). We observed a difference in cell density between the wild type and QS-deficient mutant at late log phase. This trend continued when the cultures reached the stationary phase in which the OD_600_ of the wild type was higher than 3.0 while the mutant stayed around 2.5 Figure 7.

### 3.4. Transciptome Profiling Study

**Figure 8:**
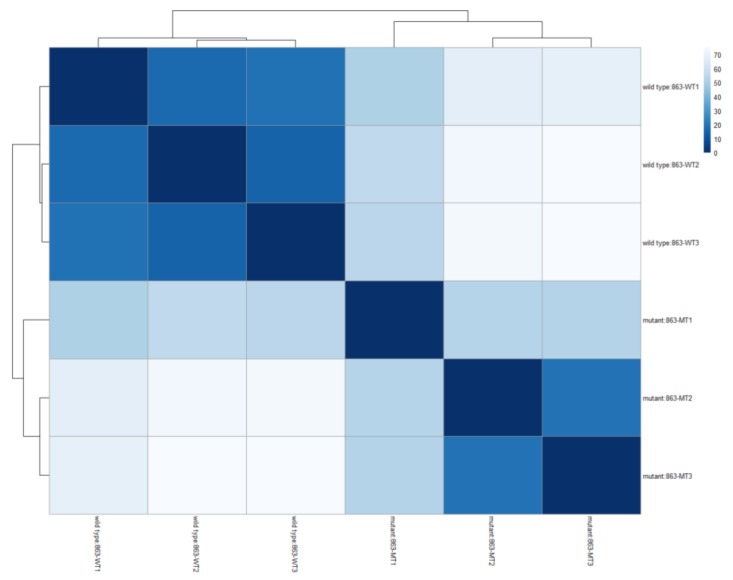
A heatmap showing sample-to-sample Euclidean distances computed from the regularized log transformation. This heat map shows that the wild type and QS-deficient mutant were clustered in respective groups, which indicates a distinct difference in the expression of various genes.

Transcriptome profiling was conducted via RNA-Seq to identify the genes regulated by QS in strain 863 and generated a total of around 43.5 million raw reads. From these total raw reads, 33.1 million (76.2%) passed the filter reads (PF read) and 98.5% of these reads were identified. The composition of every individual sample ranged from 14.6 to 19.3% of the total identified read. The heat map in Figure 8 shows both wild type and mutant strains were grouped into 2 clusters, indicating a good replication of raw data among samples.

There are a total of 352 genes found to have a differential in expression (Log_2_ |fold change| ≥ 2) between the wild type and Δ*abaI*:Km strains Table S1. Out of the 352 genes, 175 genes are downregulated while 177 genes are upregulated in the mutant strain. From this list, we found that the average magnitude of DE is significantly higher in the downregulated genes (mean Log_2_FC = −4.69) compared to the upregulated genes (mean Log_2_FC = 2.28). Table 3 shows the 10 genes with the highest magnitude in DE for both downregulated and upregulated genes.

A number of genes involved in carbon source metabolism were down-regulated in the Δ*abaI*:Km strain. The genes (A9801_RS14885, *liuA*; A9801_RS14880, *liuB*; A9801_RS14875, *liuC*; A9801_RS14870, *liuD*; A9801_RS14865, *liuE*) from the leucine/isovalerate utilization (Liu) operon were highly repressed in the mutant strain compared to the wild type. These genes had a Log_2_FC of less than −8.22 and *liuD* was the gene with the highest degree of down-regulation (Log_2_FC = −9.61) among other DE genes (Table 3). Besides, the phenylacetic acid degradation pathway was found to be affected as well. The genes (*paaH*, A9801_RS15345; *paaJ*, A9801_RS15340; *paaK*, A9801_RS15335) involved in this process were heavily down-regulated in Δ*abaI*:Km strain (Log_2_FC < −8.75).

Other than carbon source metabolism, it was found that a protein family involved in energy production was affected in the mutant strain (*A. baumannii* strain 863 Δ*abaI*:Km). The expression of a few cytochrome proteins or the components of them which are vital in the redox reaction of oxidative phosphorylation, were reduced in the mutant strain compared to the wild type. These genes included cytochrome *b* (A9801_RS14635), cytochrome *o* ubiquinol oxidase (*cyoA*, A9801_RS16360; *cyoB*, A9801_RS16365) and cytochrome *bd* complex (*cydA*, A9801_RS11155; *cydB*, A9801_RS11150). In addition, another vital component of the energy production process, F-ATPase, was also found to be affected in the mutant strain. This is because the majority of the components that constitute this protein complex were down-regulated (*atpA*, A9801_RS12840; *atpB*, A9801_RS12820; *atpD*, A9801_RS12850; *atpF*, A9801_RS12830; *atpG*, A9801_RS12845 & *atpH*, A9801_RS12835).

Interestingly, a number of genes responsible in stress response were expressed differently in the QS mutant strain. From an oxidative stress perspective, we found that two catalases (A9801_RS14960 & A9801_RS14630) were heavily repressed in the mutant strain. We also discovered that the expression of *umuC* (A9801_RS10685) and *umuD* (A9801_RS14985) are different from the wild type. These two genes encode the subunit of DNA polymerase V which is involved in a DNA repair mechanism [37]. However, both genes have an opposite pattern on one another. The first subunit, *umuD,* was down-regulated in the mutant strain while *umuC* was upregulated. In fact, *umuC* was the gene with the highest degree of differential expression among the upregulated genes (Log_2_ FC = 3.88) (Table 3).

Another group of genes worth noting is the genes associated with the ribosomes. There were 16 genes encoding for ribosomal proteins that exhibited reduced expression in the mutant strain. Seven of them (S2, A9801_RS17795; S4, A9801_RS14500; S6, A9801_RS16385; S10, A9801_RS14375; S13, A9801_RS14490; S16, A9801_RS13965 & S18, A9801_RS16390) of these genes are associated with the small subunit while the remaining 9 (L2, A9801_RS14395; L4, A9801_RS14385; L11, A9801_RS03460; L13, A9801_RS15160; L14, A9801_RS14430; L17, A9801_RS14510; L23, A9801_RS14390; L25, A9801_RS05355 & L28, A9801_RS02635) were associated with the large subunit. Besides ribosomal proteins, ribosome maturation factor, *rimM* (A9801_RS13970) and ribosome recycling factor, *rrf* (A9801_RS10865) were also repressed in *A. baumannii* strain 863 Δ*abaI*:Km.

## 4. Discussion

Whole genome sequencing was performed on strain 863 to decipher its draft genome. Raw sequencing data were processed and annotated by annotation pipelines with respective databases. From annotated data, a pair of *luxIR* homologues which consist of autoinducer synthase, *abaI* and autoinducer-binding receptor, *abaR,* was found. In this work, we aimed to characterize the AHL synthase, AbaI. From the multiple sequence alignment result, we observed that the AHL synthase protein from strain 863 possesses 10 amino acid residues which are conserved in autoinducer synthases among many bacterial species [38], and has high similarity with other *Acinetobacter* species. In addition, the phylogenetic tree shows that the autoinducer synthase protein from strain 863 was clustered with other homologues of *A. baumannii* strains. The phylogenetic tree reveals how closely related is AbaI with other autoinducer synthases in terms of molecular evolutionary distance. AbaI is clearly shown to be clustered closely among LuxI homologues of other *A. baumanii* strains, hence, strongly indicates a low rate of random mutation for this autoinducer gene. On the other hand, AbaI is the least phylogenetically related to AHL synthase from *A. oleivorans* DR1, possibly indicating that the latter was diverted in evolution. Besides, the amino acid analysis from Interproscan also showed the presence of acyl-CoA-*N*-acyltransferase domain in the putative AbaI as well as autoinducer synthase from various bacterial species that use AHLs as autoinducers.

Further studies on its upstream sequence, we managed to identify a few important promoter sequences, −10 and −35 elements. The presence of both promoter regions meets the requirement of a typical *Escherichia coli* RNA polymerase σ^70^ consensus promoter sequences. We also found a palindromic sequence that has a high possibility to be a putative *lux* box, which is associated with LuxR transcriptional binding factor, at 59 bp upstream of *abaI*. This 16-bp palindromic sequence is identical to the *lux* box found in other *Acinetobacter* species [39,40,41], indicating those species may possibly possess similar regulation of AHLs. In fact, many of these *A. baumannii* strains share a similar cluster of genes in the vicinity of *LuxI/R* genes. An interesting point is the presence of fatty acid synthesis related genes which are found at the downstream and upstream of *luxI/R* genes (Figure 4). It is highly suggested that the metabolites from these fatty acid biosynthetic machinery are used as the precursors to form the lactone ring and the acyl group of AHLs.

Following this analysis, we then determined whether strain 863 is an AHL-producing bacterium. LCMS/MS mass spectra showed the presence of hydroxyl C12 in the cell-free extract. This result correlates with other studies in which hydroxyl C12 is detected in the cultures from other *Acinetobacter* species [39,40], but not the mutant Δ*abaI*:Km strain, hence the functional role of *abaI* was confirmed in this work. Even though hydroxyl C12 is commonly produced by *Acinetobacter* species, there are exceptional cases. A study by Anbazhagan et al. [3] on multiple clinical strains of *Acinetobacter* sp. found that none of them produced hydroxyl C12. Chan et al. [9] and John et al. [42] who worked on *A. baumannii* 4KT and *A. baumannii* AB 14, respectively, also did not detect hydroxyl C12 using LCMS analysis.

As RNA-Seq provides a better result than the microarray approach, we used high throughput transcriptomic studies to investigate gene regulation on both wildtype and mutant strains [19,20,43]. In this work, we studied the transcriptome profiling of late log phase of the bacterial growth curve because we hope this phase could provide more information on QS in strain 863 during rapid growth and in preparation of the stationary phase. This is because there are studies that found that QS helps optimize growth in bacteria [44,45]. We also hope that by studying this phase, we could unlock some insights related to the differences in the growth rate and cell density in stationary phase between the wild type and QS-deficient mutant. As clearly illustrated in Figure 7, the mutant strain achieved a lower cell density at stationary phase as compared to its wildtype counterpart. This suggests that a paralyzed QS system in the mutant strain affects the basic cellular mechanism and metabolic pathways, hence producing a much slower growth rate. From the analyzed transcriptome data, we discovered that there are four distinct groups of genes which were expressed differently in the mutant strain compared to the wild type. These groups are related to carbon source metabolism, energy production, stress and ribosomes.

There are a few studies that associate QS with bacterial metabolism [46,47,48]. In this study, we observed that the expression of a number of genes related to carbon source metabolism was downregulated. The Liu operon is found to be affected the most as most of the genes in this operon were downregulated significantly in the mutant strain. The Liu operon is important in the leucine catabolism pathway [49,50]. Leucine is an alternative carbon source for metabolic activity during starvation, hence, increases the chance of survival in multiple bacterial species [51,52,53]. Therefore, in this study, we speculated that the mutant strain’s ability to survive in a nutrient-limiting environment could be hampered if the QS system was paralyzed. Nevertheless, further investigation is needed to validate such hypothesis. Besides Liu operon, a few genes in the Paa operon which are involved in phenylacetic acid degradation were also repressed in the QS-deficient mutant strain. This operon enables the bacteria to utilize phenylalanine as a carbon source and break it down into other aromatic compounds [54]. According to Bhuiyan et al. [12], phenylacetate accumulation in the infected site of zebrafish embryos could induce neutrophil chemotaxis. In our study, we found that *paaK* was greatly repressed in the mutant strain. The gene product of *paaK*, phenylacetate-CoA ligase, catalyzes the conversion of phenylacetate to phenylacetyl-CoA [55]. In fact, a *paaK* homologue which was found in *Phaeobacter gallaeciensis* DSM 17395 is essential for phenylacetate degradation [56]. Hence, we predicted that the downregulation of *paaK* could lead to phenylacetate accumulation in the infected site. However, Bhuiyan and co-workers [12] found that there was no difference in neutrophil migration pattern when the QS-deficient mutant of *A. baumannii* M2 was inoculated into zebrafish embryos. Hence, it is of high interest to further investigate the effect of downregulation of *paaK* in the mutant strain Δ*abaI*:Km.

In addition, several genes associated with energy production were affected in the QS-deficient mutant, Δ*abaI*:Km. For instance, a few genes related to cytochrome were downregulated in the QS-deficient mutant. This included cytochrome *b*, cytochrome *o* ubiquinol oxidase (*cyoA* & *cyoB*) and cytochrome *bd* complex (*cydA* & *cydB*). A study showed that the extracellular ATP production of the ∆*cyoA* mutants was lowered by more than two fold compared to the wild type strains in both *E. coli* and *Salmonella enterica.* Meanwhile, the production of extracellular ATP of the ∆*cyoB* mutant in *S. enterica* was also altered less than two fold, hence, suggesting that cytochrome *o* ubiquinol oxidase is responsible for extracellular ATP production [57]. The same study also reported that extracellular ATP could improve bacterial survivability. The cytochrome *bd* complex is a terminal oxidase that helps in establishing proton motive force. Although the biochemical reaction is less efficient, the protein’s function becomes more significant under stress conditions which are unfavorable to other terminal oxidases [58]. Boot et al. [58] also observed a growth defect in the *∆cydB* mutant of *E. coli,* which could be a factor contributing to the slower growth in the QS-deficient mutant strain 863 Δ*abaI*:Km. In *E. coli*, the cytochrome *bd* complex is important against oxidative stress from hydrogen peroxide [59]. Furthermore, multiple in vitro and in vivo studies suggested that the cytochrome *bd* complex in various bacterial species could play a role in bacterial survivability during infection in a number of vertebrate hosts [58,60,61,62,63,64,65,66].

Another important component in energy production is F-ATPase. F-ATPase generates energy in the form of ATP by transporting hydrogen ions across the cell membrane through a proton motive force [67]. Besides, F-ATPase is also reported to help bacteria to have a higher tolerance to acidic environments [68,69]. For example, *Streptococcus mutans* was found to be able to increase the expression of F-ATPase to pump hydrogen ions out of the cell to maintain its cytoplasmic pH in order to adapt to an acidic environment [70,71]. Moreover, F-ATPase can also act as ATP synthase when bacteria under starvation are exposed to an acidic environment [72]. Most of the subunits associated with the F-ATPase complex were downregulated in the QS-deficient mutant, Δ*abaI*:Km suggesting that QS could have a role in regulating these genes.

Bacteria are constantly experiencing stress in a harsh environment. Our result showed that QS is involved in some stress responses in strain 863. The synthesis of catalases was drastically downregulated in the mutant strain. This is in agreement with a report from Bhargava et al. [73] in which the QS-deficient mutant of *A. baumannii* M2 showed a reduction in catalase production and was more sensitive to hydrogen peroxide. A decrease in catalase and cytochrome *bd* complex production in the QS-deficient mutant could signify a potential disinfection approach in healthcare environments using anti-QS compounds followed by hydrogen peroxide. Abiotic stress such as oxidative stress could often lead to DNA damage [74,75]. When DNA is damaged beyond repair by other cellular mechanisms, an error-prone repair mechanism known as the “SOS response” will be triggered [37]. When the SOS response is triggered, the cell cycle is arrested and there is a marked increase in the mutation rate [76]. One of the crucial proteins involved in this mechanism is DNA Polymerase V, which consists of subunits encoded by *umuCD* [37]. The formation of DNA Polymerase V requires the binding of two RecA-mediated cleavage of UmuD and one UmuC protein [37]. Both UmuC and UmuD proteins are involved in translesion repair of DNA and are essential in induced mutagenesis. They are able to replicate DNA across DNA lesions in the presence of activated RecA [77]. In our RNA-Seq data, we discovered that the *umuC* gene was upregulated the most compared to other upregulated genes. Studies have shown that elevated *umuC* expression is generally associated with mutagenic agents such as ultraviolet rays and methyl methanesulfonate [78,79]. The action of both UmuC and UmuD proteins are needed for cell cycle checkpoint control and they are expressed in the late SOS response [80]. However, we were intrigued by our results as both *umuD* and *umuC* expression level was contradictory to each other. This phenomenon could be explained by a study done by Aranda et al. [37], which suggested that the expression of UmuC is probably regulated by UmuD which can act as a repressor or an activator. Nevertheless, further investigations are required to determine if DNA repair systems were impaired in the QS-deficient mutant.

Our result suggested that QS may play a role in strain 863 ribosome biogenesis. Multiple genes encoded for ribosomal protein were downregulated in the QS-deficient mutant. Though many ribosomal proteins are not essential in translation, these proteins could increase the integrity of ribosomes [81]. Other than protein synthesis, ribosomal proteins also act as transcriptional factors to regulate their own or other ribosomal proteins’ transcriptional level [81]. Additionally, they were also found to control transcription termination [82]. Other than ribosomal proteins, we also discovered two interesting proteins related to translation, namely, ribosome maturation factor M, RimM and ribosome recycling factor, RRF which were also downregulated in the mutant strain. Bylund et al. [83] reported that RimM plays an important role in 30S subunit maturation in *E. coli*. During the maturation process, RimM is involved in the stability of 16S 3′-domain [84,85]. Therefore, *E. coli* with deleted *rimM* had been demonstrated to have retarded growth and a lower cell density in stationary phase compared to the wild type strain [83]. In fact, it was demonstrated that *E. coli* strains from which the rimM gene has been deleted, have a sevenfold-reduced growth rate and a reduced translational efficiency [85]. Additionally, RimM was found to be critical for efficient processing of 16S rRNA. RRF encoded by *frr* is involved in the separation of ribosomes from mRNA after stop codon is detected to allow the next translation to take place [86]. A study on *E. coli* found that RRF is crucial for bacterial viability as no colony with a mutated *ffr* gene was able to be isolated unless it was complemented with a plasmid harboring intact *ffr* [86]. This could be a possible factor which contributes to the growth defect in the QS-deficient strain, as both *rimM* and *ffr* were downregulated compared to the wild type strain.

Surprisingly, *abaI* gene was not found in the top ten downregulated genes. This could be due to a very low transcription level in the wild type strain. This can be validated by the count table generated by HTSeq-count script. We found that the raw count for *abaI* gene was extremely low in the wild types among the three replicates (9, 3 and 6) while no read was found in the mutant replicates (*A. baumannii* strain 863 Δ*abaI*:Km). We postulated this result could be due to culture conditions. A recent study found that static culture condition increases the production of AHL and *abaI* expression in *A. baumannii* ATCC17978 [87]. Hence, Mayer et al. [87] speculated that the expression of QS genes in *Acinetobacter* species requires cell-to-cell or surface attachment. Nonetheless, we should not discount the differences in gene expression of other genes even with the low expression of *abaI* as demonstrated in the RNA-Seq data.

## 5. Conclusions

In this study, we had identified and characterized a *luxI* homologue, *abaI*, in multidrug-resistant strain 863. Through transcriptomic study, this paper reported that QS in strain 863 could possibly regulate more than 300 genes, and of these, four main groups of genes were significantly differentially expressed in the QS-deficient mutant, each with respective roles in bacterial fitness. We hope the findings in this study enable us to unlock more insights about the QS system in *A. baumannii,* and at the same time incorporate this knowledge in antimicrobial approaches against *A. baumannii*.

## Figures and Tables

**Table 1:** Bacteria strains and plasmids used in this study.

Strain/Plasmid	Genotype and Property	Source
Strains		
*Acinetobacter baumannii* 863	Wild type	This study
*A. baumannii* 863 Δ*abaI*:Km	Quorum sensing (QS)-deficient (*abaI* deleted) mutant	This study
Plasmids		
pKD4	Kanamycin resistant gene (Km), used as a template for selectable marker in knockout (KO) cassette	[21]
pAT04	Recombineering system (Rec_Ab_), tetracycline resistant	[21]

**Table 2:** Primers used in KO cassette construction.

Primer Name	Sequence
KO F1	AGTTACCGCCTTTCCAGCTGTTCAGTAAAGTCACTTGAAAAATAAGTGGAAGCACTTGTAATGTATGGACAGCAAGCGAACCG
KO R1	GATATGTAAAAATTAGGACTCATACCCCACGGATAGGCATGAGTCCTATATAAGAAATTATCAGAAGAACTCGTCAAGAAG
KO F2	ATACGTCATTAACCAAGTCTTCATTAAGTCAAACCTTCTCTTAGAAACCTGTAAATTCTTACAGTTACCGCCTTTCCAGC
KO R2	GAGCTATAAAAAGGATGAGACTTATTATAAGAAAGCAAACCTAAATCTAAAAACCAAGATTGATTGATATGTAAAAATTAGGAC
Screen KO F	ATACGTCATTAACCAAGTCTTCA
Screen KO R	GAGCTATAAAAAGGATGAGACT
Screen pAT04 F	GGTCTCCCCATGCGAGAGTA
Screen pAT04 R	CTCTTGCCCGGCGTCAACAC

**Table 3:** Ten Highest Fold Changes of Downregulated and Upregulated Genes in QS-Deficient Mutant.

Locus tag	Gene	Log2 FC
**Down-regulated genes**		
A9801_RS14870	3-methylcrotonyl-CoA carboxylase subunit alpha, liuD	−9.61393
A9801_RS14965	NAD(P)-dependent oxidoreductase	−9.42986
A9801_RS15335	phenylacetate-CoA ligase, paaK	−9.37778
A9801_RS14955	hypothetical protein	−9.30532
A9801_RS16540	aminoglycoside N-acetyltransferase AAC(3)-IId	−9.20034
A9801_RS15345	protein paaH	−9.18939
A9801_RS14890	TetR family transcriptional regulator	−9.1678
A9801_RS14960	catalase HPII	−9.1216
A9801_RS14875	enoyl-CoA hydratase	−9.00212
A9801_RS14855	MFS transporter	−8.94764
**Up-regulated genes**		
A9801_RS10685	UmuC	3.880526
A9801_RS13285	hypothetical protein	2.842797
A9801_RS13465	GntR family transcriptional regulator	2.737668
A9801_RS06690	2-oxo-4-hydroxy-4-carboxy-5-ureidoimidazoline decarboxylase	2.725173
A9801_RS13170	alpha/beta hydrolase	2.641788
A9801_RS01690	terminase	2.622548
A9801_RS10575	ammonium transporter	2.621587
A9801_RS16845	allantoin permease	2.61855
A9801_RS05955	MFS transporter	2.613142
A9801_RS12660	non-ribosomal peptide synthetase	2.608198

## Supplementary Materials

The following are available online at https://www.mdpi.com/2073-4425/10/4/282/s1, Table S1: List of Genes with differential expression.
