# Supplementary material for: Characterization and Transcriptome Studies of Autoinducer Synthase Gene from Multidrug Resistant Acinetobacter baumannii Strain 863
Source: Genes (Basel). 2019 Apr 8;10(4):282. doi: 10.3390/genes10040282 (PMC6523755; doi:10.3390/genes10040282)
Supplement: Supplementary file 1 [file genes-10-00282-s001.pdf]

Supplementary Material

Characterization and transcriptome studies of autoinducer synthase gene from multidrug resistant *Acinetobacter baumannii* strain 863

Chung Kiat Ng, Kah Yan How, Kok Keng Tee and Kok-Gan Chan*

***Correspondence:**

Kok-Gan Chan
kokgan@um.edu.my

Supplementary Data

Table S1 List of Genes with differential expression.

locus tag	protein id	gene	log2fc
A9801_RS14870	WP_000461798.1	3-methylcrotonyl-CoA carboxylase subunit alpha, liuD	-9.61393168
A9801_RS14965	WP_000432330.1	NAD(P)-dependent oxidoreductase	-9.42985889
A9801_RS15335	WP_000377856.1	phenylacetate-CoA ligase, paaK	-9.37778325
A9801_RS14955	WP_001145693.1	hypothetical protein	-9.30531621
A9801_RS16540	WP_000557454.1	aminoglycoside N-acetyltransferase AAC(3)-IId	-9.20034166
A9801_RS15345	WP_000130581.1	protein PaaC, paaH	-9.18939
A9801_RS14890	WP_000120691.1	TetR family transcriptional regulator	-9.16779893
A9801_RS14960	WP_000999434.1	catalase HPII	-9.1216018
A9801_RS14875	WP_000121720.1	enoyl-CoA hydratase	-9.00211661
A9801_RS14855	WP_001017546.1	MFS transporter	-8.94763637
A9801_RS15340	WP_000390391.1	acetyl-CoA acetyltransferase=3-oxoadipyl-CoA/ 3-oxo-5,6-dehydrosuberyl-CoA thiolase,paaJ	-8.75335849
A9801_RS14970	WP_000983628.1	hypothetical protein	-8.72649964
A9801_RS14940	WP_000132044.1	hypothetical protein	-8.72648616
A9801_RS14895	WP_001183739.1	fatty acid--CoA ligase	-8.66673118
A9801_RS14945	WP_000108365.1	hypothetical protein	-8.64944263
A9801_RS15330	WP_001274725.1	phenylacetic acid degradation operon negative regulatory protein PaaX	-8.58784355
A9801_RS15325	WP_001112257.1	carbonic anhydrase	-8.58566918
A9801_RS14925	WP_000251637.1	hypothetical protein	-8.52828518
A9801_RS14865	WP_001288905.1	hydroxymethylglutaryl-CoA lyase, liuE	-8.48811344
A9801_RS14880	WP_001072761.1	methylcrotonoyl-CoA carboxylase, liuB	-8.42012754
A9801_RS14975	WP_000024240.1	stress-induced protein	-8.35095081
A9801_RS14885	WP_001053639.1	isovaleryl-CoA dehydrogenase,liuA	-8.22689001
A9801_RS14980	WP_000795915.1	hypothetical protein	-7.88580114
A9801_RS16535	WP_000587837.1	hypothetical protein	-7.68416074
A9801_RS14760	WP_000501101.1	CoA transferase	-7.67732072
A9801_RS14630	WP_001061479.1	catalase	-7.63417343
A9801_RS14845	WP_001131917.1	acyl-CoA dehydrogenase	-7.54527492
A9801_RS14950	WP_000482091.1	damage-inducible protein CinA	-7.52719955

A9801_RS14765	WP_000996083.1	IclR family transcriptional regulator	-7.51211902
A9801_RS14850	WP_000995508.1	thioesterase	-7.50638428
A9801_RS14860	WP_001230726.1	LysR family transcriptional regulator	-7.38677676
A9801_RS14930	WP_001136759.1	hypothetical protein	-7.33288575
A9801_RS14985	WP_001124846.1	DNA polymerase V	-7.26552142
A9801_RS14625	WP_000670341.1	hypothetical protein	-7.25078718
A9801_RS14905	WP_000975788.1	porin	-7.04156716
A9801_RS14710	WP_000353520.1	4-hydroxybenzoate 3-monooxygenase	-6.984725
A9801_RS14775	WP_000850353.1	nitronate monooxygenase	-6.8326885
A9801_RS14900	WP_001029909.1	SAM-dependent methyltransferase	-6.81860612
A9801_RS14695	WP_001163441.1	LysR family transcriptional regulator	-6.78020246
A9801_RS15315	WP_000622491.1	hypothetical protein	-6.70654978
A9801_RS14915	WP_000119782.1	transcriptional regulator	-6.57645478
A9801_RS14665	WP_001212567.1	monooxygenase	-6.4999835
A9801_RS15310	WP_000766519.1	hypothetical protein	-6.45363755
A9801_RS14685	WP_001176552.1	NAD-dependent succinate-semialdehyde dehydrogenase	-6.40914439
A9801_RS14670	WP_000123058.1	carnitine dehydratase	-6.40658329
A9801_RS14990	WP_000885229.1	lysine transporter LysE	-6.34049561
A9801_RS14790	WP_000930800.1	acetyl-CoA acetyltransferase	-6.33979849
A9801_RS14680	WP_000750376.1	hypothetical protein	-6.23147782
A9801_RS15320	WP_001171246.1	phenylacetic acid degradation protein, paaI	-6.21643073
A9801_RS14810	WP_000617392.1	acyl-CoA dehydrogenase	-6.13180648
A9801_RS14755	WP_000617419.1	acyl-CoA dehydrogenase	-6.11169264
A9801_RS14995	WP_000376215.1	AsnC family transcriptional regulator	-6.04293153
A9801_RS14825	WP_000116039.1	3-oxoadipate CoA-transferase subunit B	-5.92741438
A9801_RS14730	WP_000930947.1	phosphonate ABC transporter permease	-5.92239929
A9801_RS14795	WP_000220487.1	3-hydroxyacyl-CoA dehydrogenase	-5.88224271
A9801_RS14745	WP_000210526.1	phosphonoacetaldehyde hydrolase	-5.84033342
A9801_RS14740	WP_000275148.1	2-aminoethylphosphonate--pyruvate transaminase	-5.82667441
A9801_RS14935	WP_000498477.1	hypothetical protein	-5.82210072
A9801_RS07170	WP_000496072.1	hypothetical protein	-5.81862558
A9801_RS14800	WP_000101935.1	3-ketoacyl-ACP reductase	-5.80242387
A9801_RS14840	WP_000445629.1	phenylacetic acid degradation protein	-5.77679186
A9801_RS14750	WP_000224800.1	MFS transporter	-5.75966692
A9801_RS07165	WP_000276191.1	hypothetical protein	-5.74395783
A9801_RS14835	WP_000411820.1	MFS transporter	-5.62599774
A9801_RS14705	WP_001139488.1	IclR family transcriptional regulator	-5.55496442
A9801_RS06105	WP_000675125.1	magnesium-translocating P-type ATPase	-5.47073938
A9801_RS14910	WP_000781116.1	lipase	-5.37950976
A9801_RS14715	WP_000434324.1	hypothetical protein	-5.3654127
A9801_RS14720	WP_000816400.1	putative 2-aminoethylphosphonate ABC transporter substrate-binding protein	-5.27123494
A9801_RS14645	WP_001049495.1	GntR family transcriptional regulator	-5.26405773
A9801_RS14830	WP_000733488.1	porin	-5.20341635

A9801_RS14770	WP_001186963.1	enoyl-CoA hydratase	-5.19696754
A9801_RS07155	WP_001090993.1	acetyl/propionyl-CoA carboxylase subuit alpha	-5.19632621
A9801_RS14660	WP_001293253.1	flavin reductase	-5.11034807
A9801_RS15305	WP_000899794.1	reverse transcriptase	-5.08990811
A9801_RS14780	WP_001990019.1	MFS transporter	-5.08273563
A9801_RS14805	WP_000116913.1	enoyl-CoA hydratase	-5.07417914
A9801_RS14635	WP_001180285.1	cytochrome b	-5.07124909
A9801_RS14815	WP_001120946.1	MFS transporter	-5.04842342
A9801_RS14820	WP_000608703.1	3-oxoadipate CoA-transferase subunit A	-5.03479834
A9801_RS14690	WP_000064100.1	4-carboxymuconolactone decarboxylase	-4.96473231
A9801_RS14700	WP_001062885.1	FMN-dependent NADH-azoreductase	-4.93934672
A9801_RS07160	WP_001211799.1	allophanate hydrolase	-4.78638075
A9801_RS14650	WP_000420415.1	peptide synthetase	-4.74544245
A9801_RS14675	WP_000081163.1	allantoin permease	-4.71254021
A9801_RS14640	WP_001277471.1	hypothetical protein	-4.69527967
A9801_RS14785	WP_000081482.1	IclR family transcriptional regulator	-4.60653902
A9801_RS14620	WP_001133075.1	TetR family transcriptional regulator	-4.51474903
A9801_RS07175	WP_000054832.1	hypothetical protein	-4.33989183
A9801_RS14725	WP_001166815.1	sulfate ABC transporter ATP-binding protein	-4.13146025
A9801_RS14655	WP_000987214.1	alpha/beta hydrolase	-4.10010331
A9801_RS06100	WP_000775740.1	methyltransferase	-3.90206192
A9801_RS14920	WP_000041623.1	hypothetical protein	-3.8902284
A9801_RS11475	WP_001084467.1	(2Fe-2S)-binding protein	-3.82831734
A9801_RS14735	WP_001057858.1	hypothetical protein	-3.67543209
A9801_RS16265	WP_000701540.1	3-hydroxyacyl-CoA dehydrogenase	-3.54485746
A9801_RS11485	WP_000815900.1	3-oxoacyl-ACP reductase	-3.46456295
A9801_RS11455	WP_001021606.1	Glu-tRNA amidotransferase	-3.45662823
A9801_RS11490	WP_000210288.1	Vanillate O-demethylase oxidoreductase	-3.38954159
A9801_RS11460	WP_001038166.1	acyl-CoA dehydrogenase	-3.37057446
A9801_RS11495	WP_001101823.1	flavin oxidoreductase	-3.16096578
A9801_RS11480	WP_001056936.1	aromatic-ring-hydroxylating dioxygenase subunit beta	-3.15681568
A9801_RS16360	WP_001250044.1	cytochrome c oxidase subunit II	-3.12224058
A9801_RS14375	WP_000070912.1	30S ribosomal protein S10	-3.05769022
A9801_RS00085	WP_001147894.1	oxidoreductase	-3.03757438
A9801_RS12835	WP_000818995.1	ATP synthase subunit delta	-2.98459937
A9801_RS00080	WP_001057073.1	acyl-CoA desaturase	-2.94882491
A9801_RS11150	WP_000575152.1	cytochrome d ubiquinol oxidase subunit II	-2.93765355
A9801_RS11655	WP_000963851.1	nucleoside-diphosphate kinase	-2.93053017
A9801_RS11465	WP_000387550.1	hypothetical protein	-2.92408855
A9801_RS15245	WP_001123845.1	hypothetical protein	-2.90472149
A9801_RS16525	WP_001330846.1	hypothetical protein	-2.87049798
A9801_RS10000	WP_001240377.1	ribonuclease P protein component	-2.69205066
A9801_RS07220	WP_001127331.1	hypothetical protein	-2.66566467

A9801_RS12915	WP_001120663.1	TonB-dependent copper receptor	-2.64516024
A9801_RS14510	WP_001216380.1	50S ribosomal protein L17	-2.62786644
A9801_RS01325	WP_000782976.1	hemerythrin	-2.62532132
A9801_RS14390	WP_001058538.1	50S ribosomal protein L23	-2.61683147
A9801_RS11470	WP_000130508.1	polyketide cyclase	-2.6117983
A9801_RS16255	WP_000853316.1	AMP-binding protein	-2.59088303
A9801_RS14490	WP_000090815.1	30S ribosomal protein S13	-2.56681411
A9801_RS11875	WP_001215920.1	acetolactate synthase small subunit	-2.56547028
A9801_RS13965	WP_000260334.1	30S ribosomal protein S16	-2.52900658
A9801_RS17645	WP_000845862.1	U32 family peptidase	-2.50235013
A9801_RS12845	WP_001284971.1	ATP synthase subunit gamma	-2.4958418
A9801_RS16385	WP_001216679.1	30S ribosomal protein S6	-2.4747047
A9801_RS14395	WP_001122317.1	50S ribosomal protein L2	-2.46690641
A9801_RS11445	WP_001076617.1	MFS transporter	-2.41320373
A9801_RS00940	WP_000065579.1	molecular chaperone GroES	-2.3927585
A9801_RS14500	WP_000135204.1	30S ribosomal protein S4	-2.38719892
A9801_RS13550	WP_000312586.1	succinyl-CoA--3-ketoacid-CoA transferase	-2.36276366
A9801_RS12920	WP_000182542.1	hypothetical protein	-2.35600071
A9801_RS12830	WP_001024694.1	F0F1 ATP synthase subunit B	-2.34760587
A9801_RS14385	WP_001050255.1	50S ribosomal protein L4	-2.33580124
A9801_RS05735	WP_000878003.1	NADH-quinone oxidoreductase subunit B	-2.3232573
A9801_RS12820	WP_000718586.1	ATP synthase subunit A	-2.31112116
A9801_RS13555	WP_001045572.1	succinyl-CoA--3-ketoacid-CoA transferase	-2.29438251
A9801_RS15160	WP_000854895.1	50S ribosomal protein L13	-2.29167082
A9801_RS14505	WP_000198631.1	DNA-directed RNA polymerase subunit alpha	-2.27114439
A9801_RS09990	WP_029423847.1	membrane protein insertase YidC	-2.26046954
A9801_RS12840	WP_001186635.1	ATP synthase subunit alpha	-2.26025365
A9801_RS05355	WP_001273421.1	50S ribosomal protein L25	-2.25784821
A9801_RS14235	WP_001164613.1	coproporphyrinogen III oxidase	-2.25764599
A9801_RS03465	WP_000832994.1	transcription termination/antitermination protein NusG	-2.24904396
A9801_RS17790	WP_000125378.1	elongation factor Ts	-2.24299455
A9801_RS05720	WP_001091095.1	NADH-quinone oxidoreductase subunit F	-2.24138473
A9801_RS17795	WP_001982118.1	30S ribosomal protein S2	-2.23592593
A9801_RS17760	WP_001177236.1	hypothetical protein	-2.22876245
A9801_RS00540	WP_000471081.1	phosphoribosylformylglycinamidine synthase	-2.22705307
A9801_RS11155	WP_000622644.1	cytochrome bd oxidase subunit I	-2.22670761
A9801_RS16390	WP_000090661.1	30S ribosomal protein S18	-2.21265564
A9801_RS12630	WP_029424600.1	N-acylhomoserine lactone synthase	-2.21016362
A9801_RS16655	WP_031981007.1	UDP-N-acetylglucosamine 4%2C6-dehydratase (inverting)	-2.2049197
A9801_RS11300	WP_000080538.1	2-dehydro-3-deoxyphosphooctonate aldolase	-2.19139754
A9801_RS12850	WP_000094481.1	ATP synthase subunit beta	-2.18600259
A9801_RS10375	WP_000833672.1	succinate dehydrogenase%2C hydrophobic membrane anchor protein	-2.17259213
A9801_RS03405	WP_000475285.1	membrane protein	-2.14402729

A9801_RS02635	WP_000048256.1	50S ribosomal protein L28	-2.13041692
A9801_RS10675	WP_000354611.1	acetyl-CoA carboxylase%2C biotin carboxyl carrier protein	-2.12808317
A9801_RS05365	WP_000952664.1	aspartate 1-decarboxylase	-2.11393182
A9801_RS14480	WP_000075233.1	preprotein translocase subunit SecY	-2.11291362
A9801_RS03460	WP_001074682.1	50S ribosomal protein L11	-2.09298769
A9801_RS04010	WP_000051669.1	preprotein translocase subunit YajC	-2.08345246
A9801_RS14430	WP_001982634.1	50S ribosomal protein L14	-2.07791111
A9801_RS13970	WP_000189236.1	ribosome maturation factor RimM	-2.07194315
A9801_RS10670	WP_000939648.1	acetyl-CoA carboxylase biotin carboxylase subunit	-2.06545
A9801_RS13975	WP_000464598.1	tRNA (guanosine(37)-N1)-methyltransferase TrmD	-2.061021
A9801_RS16260	WP_000602467.1	butyryl-CoA dehydrogenase	-2.05039725
A9801_RS03350	WP_000212712.1	acetyl-CoA C-acyltransferase FadA	-2.04865671
A9801_RS12620	WP_029424601.1	enoyl-CoA hydratase	-2.03530606
A9801_RS14135	WP_000783289.1	hypothetical protein	-2.00985735
A9801_RS10865	WP_000606428.1	ribosome recycling factor	-2.00925899
A9801_RS16365	WP_000367186.1	cytochrome ubiquinol oxidase subunit I	-2.00890207
A9801_RS08145	WP_000956372.1	hemolysin activator protein	2.00428587
A9801_RS16970	WP_000941182.1	MFS transporter	2.006360101
A9801_RS13935	WP_000546631.1	type IV pilus modification protein PilV	2.006913858
A9801_RS17775	WP_000974322.1	branched-chain amino acid ABC transporter permease	2.014909877
A9801_RS01675	WP_001108875.1	hypothetical protein	2.01689589
A9801_RS07060	WP_001000091.1	ABC transporter	2.017257409
A9801_RS01930	WP_000787149.1	hypothetical protein	2.023874264
A9801_RS17670	WP_000010367.1	nickel transporter	2.027702993
A9801_RS06670	WP_001103542.1	monooxygenase	2.028039171
A9801_RS05450	WP_000016597.1	dethiobiotin synthase	2.02928777
A9801_RS17685	WP_000010636.1	MexE family multidrug efflux RND transporter periplasmic adaptor subunit	2.029892347
A9801_RS12970	WP_001055675.1	iron transporter	2.032710922
A9801_RS03055	WP_001105725.1	amino acid transporter	2.032905185
A9801_RS04955	WP_000049453.1	hypothetical protein	2.035813521
A9801_RS04890	WP_000528177.1	DNA-binding protein	2.037377517
A9801_RS08755	WP_001051568.1	fructose 2%2C6-bisphosphatase	2.043249361
A9801_RS16200	WP_000103616.1	gamma-glutamylputrescine synthetase	2.044109788
A9801_RS13650	WP_000140381.1	hypothetical protein	2.044516871
A9801_RS07685	WP_000703023.1	hypothetical protein	2.046684933
A9801_RS01750	WP_000210194.1	antirepressor	2.052230097
A9801_RS05825	WP_000154902.1	transposase	2.061091751
A9801_RS07070	WP_000916831.1	allophanate hydrolase	2.065214267
A9801_RS04135	WP_001113293.1	hypothetical protein	2.06575196
A9801_RS03760	WP_000071770.1	SfnB family sulfur acquisition oxidoreductase	2.068444435
A9801_RS08040	WP_000482354.1	hypothetical protein	2.070154928
A9801_RS06270	WP_000770091.1	hypothetical protein	2.075552547
A9801_RS04885	WP_000160878.1	NADPH-dependent ferric siderophore reductase	2.076029443

A9801_RS07395	WP_000648017.1	MFS transporter	2.076089477
A9801_RS08140	WP_001180448.1	TetR family transcriptional regulator	2.076147263
A9801_RS09580	WP_000431603.1	branched-chain amino acid ABC transporter permease	2.081553772
A9801_RS03990	WP_001988076.1	hypothetical protein	2.084567336
A9801_RS02300	WP_000926498.1	short-chain dehydrogenase	2.084805611
A9801_RS02900	WP_000375494.1	LysR family transcriptional regulator	2.089693164
A9801_RS07105	WP_000217385.1	urea carboxylase	2.092590283
A9801_RS16775	WP_001173274.1	sulfonate ABC transporter substrate-binding protein	2.094619846
A9801_RS12560	WP_001247568.1	AsnC family transcriptional regulator	2.094647463
A9801_RS06555	WP_001191147.1	hypothetical protein	2.097909392
A9801_RS00460	WP_001048807.1	sulfate ABC transporter permease subunit CysW	2.099053013
A9801_RS05015	WP_001001107.1	amino acid oxidase	2.099417493
A9801_RS13225	WP_000091115.1	saccharopine dehydrogenase	2.102900311
A9801_RS04875	WP_000893003.1	acinetobactin biosynthesis protein	2.103520003
A9801_RS01485	WP_000644001.1	DNA polymerase III subunit epsilon	2.112364355
A9801_RS03645	WP_000898893.1	pilus assembly protein	2.115955901
A9801_RS05925	WP_000807285.1	lysine transporter LysE	2.117495245
A9801_RS13455	WP_000771649.1	FMN-dependent NADH-azoreductase	2.122334687
A9801_RS08685	WP_000442394.1	MFS transporter	2.130606362
A9801_RS11580	WP_000345069.1	MFS transporter	2.131714316
A9801_RS12990	WP_000965123.1	permease	2.13192382
A9801_RS09505	WP_001061070.1	permease	2.135422089
A9801_RS17690	WP_001027056.1	multidrug efflux RND transporter permease subunit	2.140665862
A9801_RS02560	WP_000137990.1	phosphatase	2.14233468
A9801_RS02350	WP_000875353.1	arylsulfatase	2.145120871
A9801_RS04810	WP_001095752.1	ABC transporter	2.145512985
A9801_RS04835	WP_000744381.1	#N/A	2.145779743
A9801_RS02345	WP_000769049.1	sulfatase-modifying protein	2.147068981
A9801_RS14605	WP_000013374.1	GGDEF domain-containing protein	2.148050862
A9801_RS11510	WP_000792905.1	hypothetical protein	2.151412684
A9801_RS07995	WP_000181290.1	acyl-CoA synthetase	2.153971548
A9801_RS13105	WP_001044478.1	hypothetical protein	2.15653849
A9801_RS04840	WP_001177743.1	peptide synthetase	2.157654142
A9801_RS01790	WP_000190165.1	hypothetical protein	2.157869437
A9801_RS13095	WP_000841495.1	hypothetical protein	2.162087489
A9801_RS12635	WP_064987440.1	DUF4902 domain-containing protein	2.16362884
A9801_RS12645	WP_029424599.1	acyl-CoA synthetase	2.165744932
A9801_RS01850	WP_000030337.1	hypothetical protein	2.167567851
A9801_RS00855	WP_001133239.1	TetR family transcriptional regulator	2.16776161
A9801_RS17095	WP_001066272.1	nitrate reductase	2.171355085
A9801_RS01655	WP_000371258.1	hypothetical protein	2.171397815
A9801_RS07850	WP_000539749.1	hypothetical protein	2.174304747
A9801_RS16910	WP_000195658.1	serine acetyltransferase	2.175315299

A9801_RS04825	WP_000603876.1	histidine decarboxylase	2.176174658
A9801_RS13940	WP_000079200.1	pilus assembly protein PilW	2.176940942
A9801_RS15925	WP_064987443.1	MFS transporter	2.177640771
A9801_RS16875	WP_001130362.1	glutamine ABC transporter ATP-binding protein	2.177969984
A9801_RS05975	WP_000994352.1	tricarballylate utilization protein B	2.178510009
A9801_RS05745	WP_001147032.1	diguanylate cyclase	2.180459818
A9801_RS16810	WP_000828360.1	C4-dicarboxylate ABC transporter	2.18201413
A9801_RS08195	WP_000366420.1	LysR family transcriptional regulator	2.182400302
A9801_RS10580	WP_000184466.1	hypothetical protein	2.186376511
A9801_RS13470	WP_000448792.1	fumarate reductase/succinate dehydrogenase flavoprotein subunit	2.187834453
A9801_RS15940	WP_001011888.1	fumarylacetoacetate hydrolase	2.19238627
A9801_RS15595	WP_000392676.1	membrane protein	2.192597114
A9801_RS15960	WP_001026282.1	MFS transporter	2.198361973
A9801_RS04815	WP_001281538.1	ABC transporter	2.202520831
A9801_RS08780	WP_000971116.1	hypothetical protein	2.207359655
A9801_RS04845	WP_000717759.1	putative histamine N-monooxygenase	2.215169681
A9801_RS11585	WP_001165785.1	GguC protein	2.215864163
A9801_RS02305	WP_000381874.1	MFS transporter	2.223234947
A9801_RS01780	WP_001167468.1	hypothetical protein	2.225187605
A9801_RS01550	WP_000218432.1	hypothetical protein	2.22759193
A9801_RS17220	WP_000437831.1	hypothetical protein	2.231765093
A9801_RS09825	WP_000845026.1	class II aldolase	2.233518681
A9801_RS04930	WP_001076001.1	MFS transporter	2.238749689
A9801_RS09480	WP_000792700.1	TetR family transcriptional regulator	2.240964401
A9801_RS12965	WP_000770288.1	iron transporter	2.256081822
A9801_RS02075	WP_001271333.1	arginine:ornithine antiporter	2.274354586
A9801_RS01640	WP_001166851.1	hypothetical protein	2.274881367
A9801_RS02550	WP_001072722.1	twin arginine-targeting protein translocase TatC	2.27496096
A9801_RS04870	WP_001210787.1	iron ABC transporter permease	2.277179235
A9801_RS17290	WP_000429471.1	threonine transporter	2.286306553
A9801_RS02030	WP_002029060.1	receptor protein	2.287528002
A9801_RS16945	WP_000102767.1	murein hydrolase transporter LrgA	2.287733088
A9801_RS03390	WP_002001381.1	hypothetical protein	2.288014042
A9801_RS13055	WP_000243697.1	hypothetical protein	2.290254866
A9801_RS04350	WP_000457787.1	quaternary ammonium transporter	2.296989196
A9801_RS02020	WP_000583863.1	RNA polymerase sigma factor	2.297427261
A9801_RS16895	WP_000267781.1	lysine transporter LysE	2.299183278
A9801_RS13825	WP_000695065.1	hypothetical protein	2.309069025
A9801_RS00735	WP_000494036.1	polysaccharide biosynthesis protein GtrA	2.315987142
A9801_RS13930	WP_001214061.1	prepilin-type N-terminal cleavage/methylation domain-containing protein	2.318767732
A9801_RS15395	WP_000203775.1	aspartate:proton symporter	2.319685295
A9801_RS17665	WP_000569997.1	histidine/lysine/arginine/ornithine ABC transporter permease HisM	2.326645723
A9801_RS17720	WP_000896943.1	hypothetical protein	2.329179022

A9801_RS02255	WP_000753775.1	hypothetical protein	2.334128784
A9801_RS10700	WP_000114559.1	hypothetical protein	2.334746266
A9801_RS17060	WP_000107057.1	MFS transporter	2.336206084
A9801_RS02140	WP_000267735.1	C4-dicarboxylate ABC transporter	2.337797306
A9801_RS05025	WP_001120607.1	TetR family transcriptional regulator	2.341866417
A9801_RS13945	WP_000149374.1	hypothetical protein	2.34664335
A9801_RS11575	WP_001060816.1	dihydroxy-acid dehydratase	2.349643768
A9801_RS04905	WP_001133249.1	TetR family transcriptional regulator	2.352195969
A9801_RS10690	WP_000039930.1	nitrate transporter	2.35532519
A9801_RS16765	WP_000724539.1	hypothetical protein	2.358102508
A9801_RS17165	WP_000269038.1	transposase	2.35874047
A9801_RS16790	WP_029424622.1	ABC transporter permease	2.372513905
A9801_RS12555	WP_000051099.1	hypothetical protein	2.380981055
A9801_RS00725	WP_001140944.1	hypothetical protein	2.38530267
A9801_RS04855	WP_001104139.1	ferric anguibactin-binding protein	2.385667372
A9801_RS03730	WP_000264517.1	porin	2.389960257
A9801_RS17205	WP_000792950.1	TetR family transcriptional regulator	2.392070891
A9801_RS00455	WP_000083115.1	sulfate ABC transporter permease subunit CysT	2.398417327
A9801_RS05900	WP_001989618.1	TetR family transcriptional regulator	2.401091324
A9801_RS15460	WP_000107496.1	TetR family transcriptional regulator	2.402229683
A9801_RS17910	WP_000464149.1	hypothetical protein	2.416695527
A9801_RS03650	WP_001078509.1	fimbrial protein	2.426050941
A9801_RS06370	WP_002046352.1	MATE efflux family protein	2.427729176
A9801_RS17770	WP_000118659.1	hypothetical protein	2.430991858
A9801_RS02045	WP_000837763.1	biopolymer transporter TonB	2.435972979
A9801_RS02040	WP_000831634.1	peptide signal protein	2.447374506
A9801_RS03850	WP_000451663.1	amino acid transporter	2.461079475
A9801_RS07110	WP_001090541.1	urea carboxylase	2.462173052
A9801_RS10695	WP_001125255.1	hypothetical protein	2.462286833
A9801_RS09515	WP_001089008.1	MFS transporter	2.46399091
A9801_RS02005	WP_000182783.1	MFS transporter	2.466358681
A9801_RS16785	WP_000689552.1	alkanesulfonate monooxygenase	2.47198802
A9801_RS16035	WP_001075435.1	ethanolamine permease	2.479551341
A9801_RS16950	WP_001028710.1	membrane protein	2.479735874
A9801_RS01475	WP_031944393.1	hypothetical protein	2.482019871
A9801_RS02905	WP_000207252.1	short chain dehydrogenase	2.496541914
A9801_RS15405	WP_000214022.1	MFS transporter	2.497101825
A9801_RS12870	WP_000786954.1	AraC family transcriptional regulator	2.503140446
A9801_RS10600	WP_000632986.1	phospholipase C%2C phosphocholine-specific	2.510199022
A9801_RS16780	WP_029424621.1	sulfonate ABC transporter substrate-binding protein	2.520162422
A9801_RS13485	WP_000096311.1	ABC transporter permease	2.520737467
A9801_RS16225	WP_000891197.1	potassium-transporting ATPase subunit KdpA	2.534667356
A9801_RS00165	WP_002016789.1	MFS transporter	2.537413701

A9801_RS13815	WP_000201227.1	membrane protein	2.546251152
A9801_RS17090	WP_000694034.1	Asp/Glu racemase	2.556473084
A9801_RS04865	WP_001223274.1	iron ABC transporter permease	2.562207831
A9801_RS16865	WP_001104463.1	amino acid ABC transporter	2.57530528
A9801_RS09945	WP_029423852.1	(2Fe-2S)-binding protein	2.577230752
A9801_RS08200	WP_001166494.1	hypothetical protein	2.581607644
A9801_RS12180	WP_000918635.1	carboxymethylenebutenolidase	2.589452658
A9801_RS12650	WP_029424598.1	acyl-CoA dehydrogenase	2.593898901
A9801_RS12660	WP_029424597.1	non-ribosomal peptide synthetase	2.608198154
A9801_RS05955	WP_000383637.1	MFS transporter	2.613142109
A9801_RS16845	WP_000116422.1	allantoin permease	2.618549889
A9801_RS10575	WP_002001094.1	ammonium transporter	2.621586709
A9801_RS01690	WP_001162227.1	terminase	2.622548316
A9801_RS13170	WP_000520419.1	alpha/beta hydrolase	2.641788046
A9801_RS06690	WP_000477669.1	2-oxo-4-hydroxy-4-carboxy-5-ureidoimidazoline decarboxylase	2.725172763
A9801_RS13465	WP_000080817.1	GntR family transcriptional regulator	2.737667985
A9801_RS13285	WP_001178921.1	hypothetical protein	2.842796549
A9801_RS10685	WP_000652023.1	UmuC	3.880526037
